# The impact of teacher training on the evaluation and selection of STEM augmented reality applications and TPACK self-assessment

**DOI:** 10.3389/fpsyg.2025.1657028

**Published:** 2025-12-16

**Authors:** Janine Küng, Dorothee Brovelli

**Affiliations:** Institute for STEM Education and Sustainability, University of Teacher Education Lucerne, Lucerne, Switzerland

**Keywords:** TPACK, teacher training, teacher education, STEM, augmented reality, AR, digital competencies, digital transformation

## Abstract

**Introduction:**

The digital transformation of education is reshaping the demands placed on teachers, as new competencies are required for the integration of emerging technologies such as augmented reality (AR). To utilize such digital learning resources effectively, teachers must initially be able to evaluate and select them based on their professional knowledge, which the TPACK framework conceptualizes by integrating technological, pedagogical, and content knowledge. However, it remains an open question to what extent teacher training supports the development of these competencies.

**Methods:**

This study examines how teacher training influences prospective teachers' knowledge-based evaluation and selection of STEM-related AR applications, as well as their self-assessed digital competencies. A total of *N* = 305 prospective lower secondary school teachers evaluated two AR applications related to one of three STEM topics, selected the one they considered more suitable for use in the classroom and provided self-assessments of their PCK, TPK and TPACK. To explore potential differences based on training level, comparisons were made between undergraduate and graduate students. The data were analyzed using qualitative content analysis, Mann-Whitney U tests, and Pearson chi-squared tests.

**Results:**

Graduate students placed greater emphasis on TCK when evaluating the mathematics (*p* = 0.002, *d* = −0.58) and the physics AR applications (*p* < 0.001, *d* = −0.65), whereas undergraduates focused more on TPK in these subject assessments (mathematics: *p* = 0.007, *d* = 0.50; physics: *p* = 0.018, *d* = 0.43). Additional differences appeared within subject assessments, with the strongest effect observed for the PCK subcategory of model knowledge and use in the physics assessment (*p* < 0.001, *d* = −0.85). Under ideal conditions, undergraduates showed stronger preferences in selecting AR application in the mathematics (*p* = 0.030, Cramér's *V* = 0.195) and the biology assessment (*p* = 0.004, Cramér's *V* = 0.262), while graduates demonstrated a more balanced selection pattern. Graduates rated their PCK higher overall (*p* = 0.002, *d* = −0.37), whereas no significant group differences were observed in self-assessed TPK or TPACK.

**Discussion:**

The findings show changes in the knowledge-based evaluation and selection of AR applications, as well as in self-assessed PCK during teacher training. However, improvements in both the reference to TPACK and self-assessment of TPACK were less pronounced, indicating room for further development. This aligns with prior research suggesting that more comprehensive, model-based approaches (e.g., SQD) and stronger role modeling by teacher educators could better support teachers in effectively integrating digital resources like AR.

## 1 Introduction

### 1.1 Conditions for effective integration of digital learning resources

The digital transformation of education has led to an increasing use of digital learning resources from both formal and informal sources, which often differ considerably in quality (OECD, 2023; Sailer et al., 2021). Therefore, it is becoming increasingly important to understand the factors that promote effective integration of digital learning resources. In this context, the Will, Skill, Tool (WST) model by Knezek and Christensen (2016) is a useful framework, as it identifies three central conditions for effective technology use in education: a positive attitude toward using technology (Will), the necessary skills to use technology effectively (Skill), and sufficient access to technological infrastructure and resources (Tool). Regarding the skill component, teachers must be able to evaluate the quality of digital learning resources, make a knowledge-based selection and incorporate them into their teaching in a way that is both pedagogically meaningful and content-appropriate (Woltran et al., 2022; Gonscherowski et al., 2025).

### 1.2 Theoretical perspectives on teachers' digital competence

**Figure 1:**
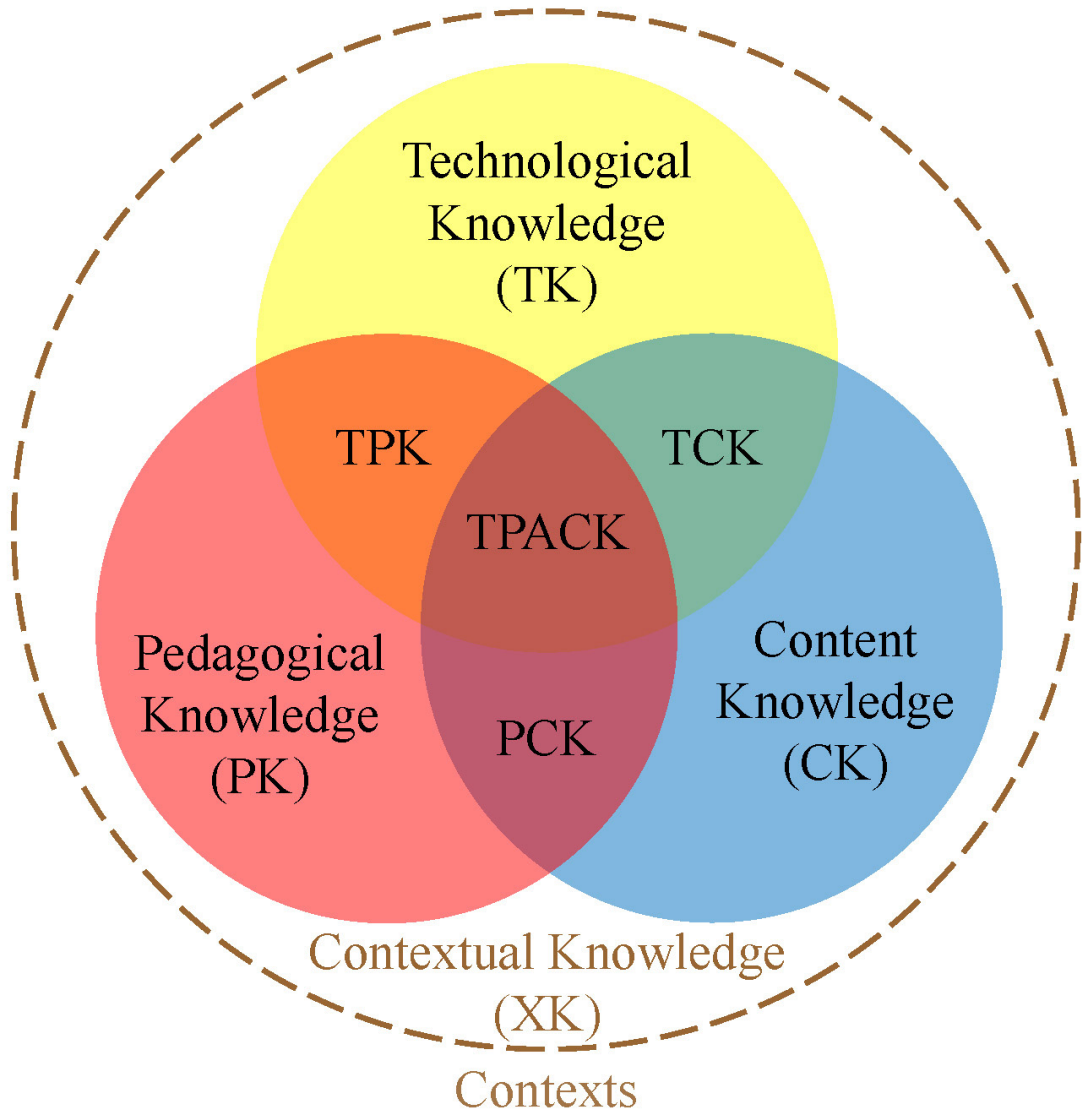
TPACK model based on Koehler and Mishra (2009), adapted by Petko et al. (2025), own illustration.

To model the necessary competencies for effective integration of digital learning resources, Koehler and Mishra (2009) expanded Shulman's (1987) pedagogical content knowledge (PCK) model by incorporating four additional technological knowledge domains. The resulting technological pedagogical content knowledge (TPACK) framework includes technological knowledge (TK), technological content knowledge (TCK) and technological pedagogical knowledge (TPK), as well as the integrative domain of technological pedagogical content knowledge (TPACK). Since its introduction, the TPACK framework has undergone substantial refinement, particularly regarding the role of contextual factors (e.g. Brianza et al., 2022). A recent conceptual synthesis by Petko et al. (2025) integrates two central perspectives on context within the TPACK framework: external contextual influences and contextual knowledge (XK) as a distinct knowledge domain (see Figure 1). Both aspects are recognized as complementary and equally essential. The TPACK model emphasizes that teacher education and research should focus on the pedagogical application of digital technologies to support subject-specific learning goals, rather than merely focusing on general technological skills (Koehler and Mishra, 2009; Schmid et al., 2020b). TPACK is often assessed through self-report measures (Akyuz, 2018; Mohammadpour and Maroofi, 2025). However, comparative studies suggest that self-assessments tend to be only partially aligned with more objective methods, such as performance-based assessments or classroom observations (Stinken-Rösner et al., 2023; Mohammadpour and Maroofi, 2025).

**Figure 2:**
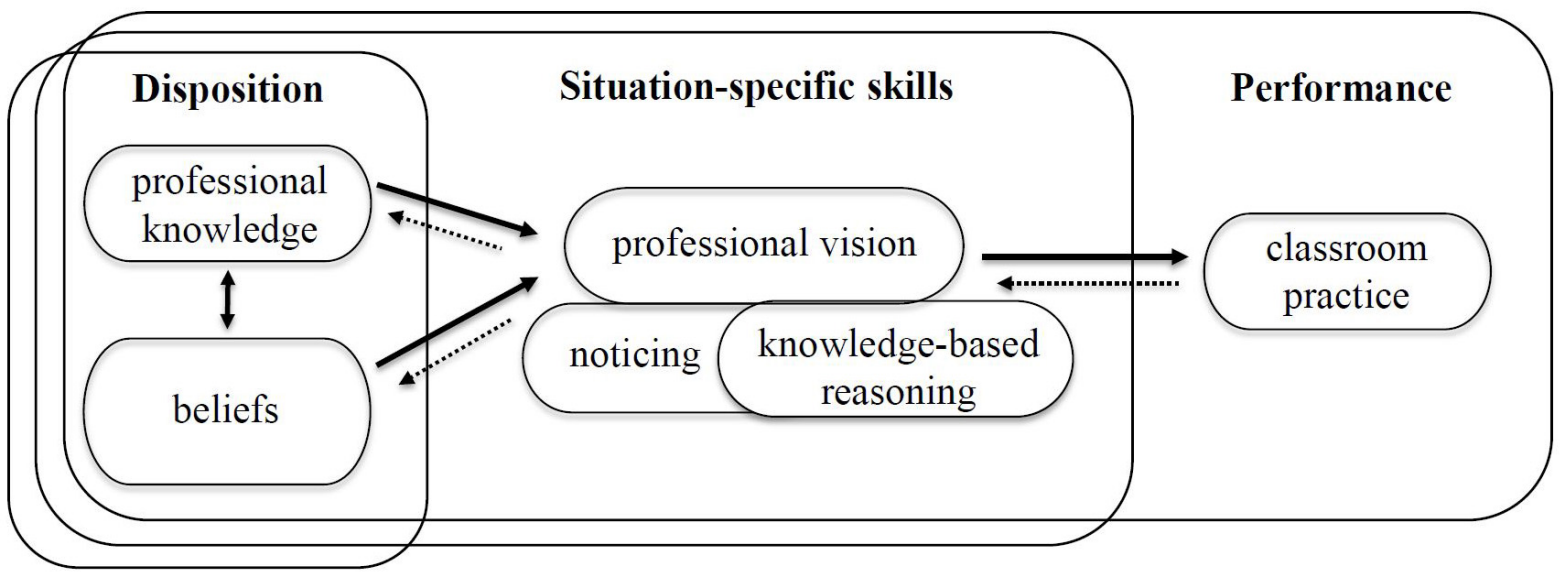
PID model from Blömeke et al. (2015), adapted by Meschede et al. (2017), own illustration.

Although Koehler and Mishra's (2009) original TPACK framework encompasses both knowledge and skills, the term “knowledge” is predominantly used throughout literature. In the context of teacher education, this terminology may be limiting, as the broader concept of competence offers a more comprehensive perspective. One widely adopted competency model is the perception-interpretation-decision-making (PID) framework by Blömeke et al. (2015), which Meschede et al. (2017) linked to the concept of professional vision (van Es and Sherin, 2008; see Figure 2). This emphasizes the central role of cognitive processes in classroom situations. Professional vision comprises two interrelated components: noticing, which is the ability to identify relevant events in the classroom, and knowledge-based reasoning, which is the ability to interpret these events using professional knowledge. Professional vision is considered crucial for adaptive teaching (Meschede et al., 2017) and is typically assessed using text- or video-based classroom vignettes (Weyers et al., 2023; Atanasova et al., 2023).

### 1.3 Fostering digital competencies in teacher education

The growing incorporation of digital learning resources into educational practice highlights the importance of systematically developing digital competencies among teachers. Teacher education programs play a central role in this endeavor, as they are responsible for creating learning environments that not only build technological knowledge but also enable teachers to use digital learning resources effectively (Tondeur et al., 2021; Lindfors et al., 2021). One well-established framework that can guide this effort is the Synthesis of Qualitative Evidence (SQD) model, which was developed by Tondeur et al. (2012). Based on 19 qualitative studies, the SQD model identified six key teaching strategies for developing digital competence in teacher education: teacher educators acting as role models, opportunities for reflection, learning by design, collaboration, authentic learning experiences, and continuous feedback. Many interventions aimed at promoting digital competencies in teacher education are grounded on this model (e.g. Lachner et al., 2021; Weiler et al., 2022). Recently Tondeur et al. (2025) suggested an updated SQD-model (SQD2) which introduces new themes, including “Digital Identity”, “Instructional Design Models”, and “Affective Dimensions”.

In addition to the SQD model, the TPACK model is also frequently used to design courses that promote digital competencies. However, it is important to note that the SQD and the TPACK model operate at different conceptual levels: while SQD outlines practical strategies and conditions for effective professional learning, TPACK describes the specific knowledge domains teachers need to integrate technology meaningfully. Because TPACK is a theoretical model and does not provide concrete design principles like SQD, interventions based on TPACK tend to vary considerably in their focus and implementation. Fabian et al. (2024) found that most programs emphasize technological knowledge (TK), while over 20% of the considered interventions did not explicitly address the integrative domain of technological pedagogical content knowledge (TPACK). In addition, positive associations between CK, PCK, TCK, and TPACK were found which indicates that TPACK is typically considered within the context of specific subjects.

Meta-analyses by Ning et al. (2022) and Fabian et al. (2024) support the effectiveness of interventions to foster teachers' digital competencies in teacher education. Such programs can positively influence prospective teachers' technological pedagogical content knowledge (TPACK). But there seems to be a considerable gap between research and practice. Studies show that many teacher educators themselves exhibit relatively low levels of professional digital competence and that digital technologies remain insufficiently embedded in teacher education programs (Tondeur et al., 2019; Voithofer and Nelson, 2020; Lindfors et al., 2021).

### 1.4 Current state of teachers' digital competencies

Empirical studies of prospective teachers' digital competencies suggest that both early-stage and advanced prospective teachers tend to demonstrate lower levels of digital competence than students in other academic disciplines. This discrepancy is particularly pronounced among those who do not specialize in STEM subjects. Furthermore, a significant proportion of prospective teachers fail to meet established baseline standards for digital competence (Senkbeil et al., 2021; Johnson et al., 2023).

In a German study, Zinn et al. (2022) found that prospective teachers rated their knowledge of technology-related domains (TK, TCK, TPK and TPACK) as moderate. While significant improvements in self-assessment were observed in general professional knowledge areas, specifically content knowledge (CK), pedagogical knowledge (PK), and pedagogical content knowledge (PCK), changes in technology-related domains were either marginal or statistically insignificant. Notably, there was greater variability in the self-reported technology-related knowledge, suggesting that prospective teachers have different levels of digital competence. Similarly, the German study by Weidlich and Kalz (2023) found no substantial gains in prospective teachers' self-assessed competencies within technology-related TPACK domains over the course of their studies. In a Finnish study, Valtonen et al. (2019) observed an increase in self-assessment scores for all TPACK areas during teacher education. The most pronounced growth occurred in pedagogy-related domains (PCK, PK, and TPACK), while improvements in CK and TK remained modest. A Chinese study reported generally high self-ratings of TPACK among prospective teachers, with TPK and PK rated highest and TK lowest. Significant differences emerged between different training stages for CK, PK, TPK, TCK, and TPACK, but not for TK and PCK (Li et al., 2022).

### 1.5 Teacher competencies in the use of augmented reality

Considering the increasing significance of digital competency development in teacher education, it is crucial to explore how these skills are implemented in practice with specific technologies. One such technology is augmented reality (AR), which overlays digital information and objects onto the real world using smartphones, tablets, or AR glasses (Azuma, 1997). Research suggests that there are many advantages to using AR for education. For example, it can enhance students learning motivation and learning achievement (Howard and Davis, 2023; Jiang et al., 2025; Chang et al., 2022). AR enables the visualization of complex and abstract concepts through interactive, three-dimensional models (Koumpouros, 2024; Jiang et al., 2025). This is particularly valuable for illustrating structures and processes that are difficult to represent using static, two-dimensional media, such as the anatomy and function of the human heart. However, studies on AR's impact on cognitive load yield mixed results. While some studies suggest that AR may increase cognitive load due to the complexity of the information presented in multiple modalities, others find that it can reduce cognitive load by making abstract content more tangible and accessible (AlGerafi et al., 2023; Poupard et al., 2024; Buchner et al., 2021).

Despite its potential, AR is still rarely used in schools. This limited adoption is primarily due to a lack of high-quality educational AR applications, technical limitations, and restrictive policies on device usage (Radu et al., 2022; Tan et al., 2023). Furthermore, many (prospective) teachers have limited hands-on experience with AR and are often only familiar with it through gaming applications such as Pokémon Go. AR also seems to be rarely incorporated into teacher training programs (Schwaiger et al., 2024; Trust et al., 2021; Avila-Garzon et al., 2021). While research shows that (prospective) teachers generally have a positive attitude toward using AR in the classroom, they often lack confidence in creating and managing AR applications (Nikou et al., 2024; Zgraggen, 2023; Perifanou et al., 2023). In the context of AR, teachers tend to rate the foundational TPACK domains, content knowledge (CK), technological knowledge (TK) and pedagogical knowledge (PK), higher than the integrative domains, particularly technological pedagogical knowledge (TPK). For instance, language teacher candidates reported low confidence in selecting appropriate AR teaching methods (Belda-Medina and Calvo-Ferrer, 2022). Similarly, in a professional development program, physics teachers designed AR experiments which were then evaluated by experts, who rated the teachers' technological pedagogical knowledge (TPK) as the weakest among the assessed competencies (Freese et al., 2023).

### 1.6 Research gaps and research questions

Despite the growing body of research on the digital competencies of prospective teachers (Basilotta-Gómez-Pablos et al., 2022), important gaps remain which this study aims to address. While studies from countries such as Germany, Finland, and China have investigated the development of TPACK domains during teacher training (Zinn et al., 2022; Weidlich and Kalz, 2023; Valtonen et al., 2019; Li et al., 2022), there is, to the best of the authors' knowledge, no equivalent research that focused on the Swiss context. This is noteworthy, given that national differences in the structure and content of teacher education, such as the emphasis placed on content knowledge (CK) vs. pedagogical content knowledge (PCK), significantly influence the development of competencies (e.g. Kleickmann et al., 2012). Therefore, a key objective of this study is to explore whether similar developmental patterns of TPACK during teacher education exist in Switzerland or if distinct trajectories emerge.

In addition to this geographical gap, existing literature often adopts a generalized and decontextualized perspective on TPACK (Greene and Jones, 2020; Petko et al., 2025). However, the TPACK framework explicitly emphasizes the situated integration of technological, pedagogical, and content knowledge within specific disciplinary and technological contexts (see section 1.2; Koehler and Mishra, 2009; Schmid et al., 2020b; Brianza et al., 2022). To address this conceptual limitation, the present study adopts a subject- and technology-specific lens. Six AR applications spanning three distinct STEM topics were selected to enable meaningful comparisons while accounting for the unique content and pedagogical demands of each field. The study focuses on augmented reality (AR) due to its innovative potential, particularly in STEM education (e.g., Jiang et al., 2025), and limited exposure among prospective teachers (Schwaiger et al., 2024; Trust et al., 2021; Avila-Garzon et al., 2021). This focus allows for an investigation of how prospective teachers adapt and transfer their knowledge to novel technologies within subject-specific instructional contexts.

Furthermore, previous studies have often relied on self-assessments to measure TPACK development (Akyuz, 2018; Mohammadpour and Maroofi, 2025), which provide limited insight into the actual application of knowledge (Stinken-Rösner et al., 2023; Mohammadpour and Maroofi, 2025). To address this methodological limitation, the present study employs a vignette-based design inspired by the assessment of professional vision (see section 1.2; Weyers et al., 2023; Atanasova et al., 2023) to analyze which TPACK areas prospective teachers utilize when evaluating and selecting AR applications. To this end, the textual responses provided by the prospective teachers during the evaluation of the AR applications were analyzed using qualitative content analysis, and their application selection was systematically recorded and quantified. Despite their limitations, self-assessments of PCK, TPK, and TPACK are included as a complementary measure to these professional vision-based evaluation and selection tasks, offering valuable insights into teachers' perceived competencies and facilitating comparison with prior research that used self-report measures. This combined approach enables a more comprehensive understanding of prospective teachers' competencies by including both perceived competence and demonstrated reasoning in authentic evaluation and decision-making scenarios.

This study aims to shed light on which aspects of TPACK are already being fostered in Swiss teacher education programs and where improvements could be targeted. To capture competence development during teacher training, the study compares undergraduate and graduate students preparing to teach STEM subjects at lower secondary level. The research questions are as follows:

RQ1: How do undergraduate and graduate students of lower secondary STEM education differ in their reference to TPACK areas and their subtopics when evaluating augmented reality applications for STEM teaching?
RQ2: How do undergraduate and graduate students of lower secondary STEM education differ in their selection of augmented reality applications for STEM teaching?
RQ3: How do undergraduate and graduate students of lower secondary STEM education differ in their self-assessment of pedagogical content knowledge (PCK), technological pedagogical knowledge (TPK) and technological pedagogical content knowledge (TPACK)?

To structure the analysis and presentation of the findings, the remainder of this article is organized as follows: Section 2 outlines the materials and methods, including the study framework, sample, and data analysis procedures. Section 3 presents the results for each research question. Section 4 provides an in-depth discussion of the findings and is followed by the conclusion and implications in Section 5 and the limitations and research outlook in Section 6.

## 2 Materials and methods

### 2.1 Study framework

The study is based on the methodological approach of vignette studies, which are often used to investigate professional vision (see Section 1.2; Weyers et al., 2023; Atanasova et al., 2023). Instead of evaluating descriptions or videos of classroom situations, this study simulated a planning scenario in which prospective lower secondary school teachers were tasked with testing and evaluating two augmented reality (AR) applications for a STEM subject, selecting one of them and incorporating it into a lesson plan. The evaluation was carried out on site using an online questionnaire. Since predefined items in professional vision tests can influence participants' attention (e.g. Dückers et al., 2022), an open response format was chosen for all application assessment prompts. First, the prospective teachers tested each application and provided an initial evaluation by identifying positive, neutral and negative aspects. Then they were asked to choose one of the two applications under ideal conditions (suitable class level, tablets and accessories available, etc.) and justify their choice. Next, the prospective teachers were asked to describe how they would use their chosen application for STEM teaching under ideal conditions. Then, considering real-world conditions (everyday teaching context), they were asked to select one, both or none of the applications. Lastly, they described the extent to which planning with the chosen augmented reality application(s) under ideal conditions would differ from planning under real-world conditions. The exact wording of the individual prompts can be found in the Supplementary material.

Six AR applications covering three distinct STEM topics (see Table 1) were selected to allow for cross-subject and cross-application insights into the focus of prospective undergraduate and graduate teachers when evaluating and selecting augmented reality applications. Each pair of applications addressed a comparable topic to enable meaningful comparisons and informed selection decisions. All selected applications had to be suitable for lower secondary education, ensuring adequate usability and content relevance. The applications were identified through an extensive search of scientific publications, app stores, and search engines. As this search revealed a shortage of high-quality AR applications appropriate for lower secondary education, some applications were translated and others newly developed or adapted in collaboration with Lucerne University of Applied Sciences and Arts (HSLU) to meet the study's requirements. Due to the time required to test each application, participants assessed only two applications per session. However, some prospective teachers participated in the study more than once (see Section 2.2.2).

**Table 1:** AR applications used in the study.

**Physics**	**Biology**	**Mathematics**
AR circuit constructor	Blood group compatibility	Sólidos RA
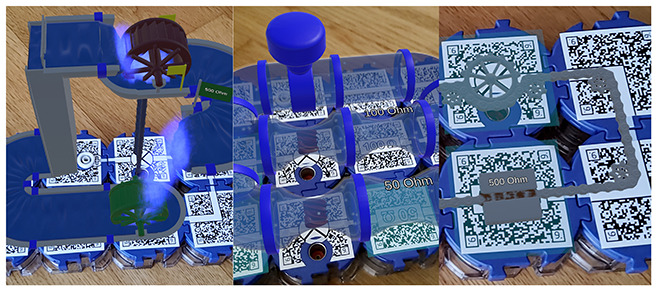	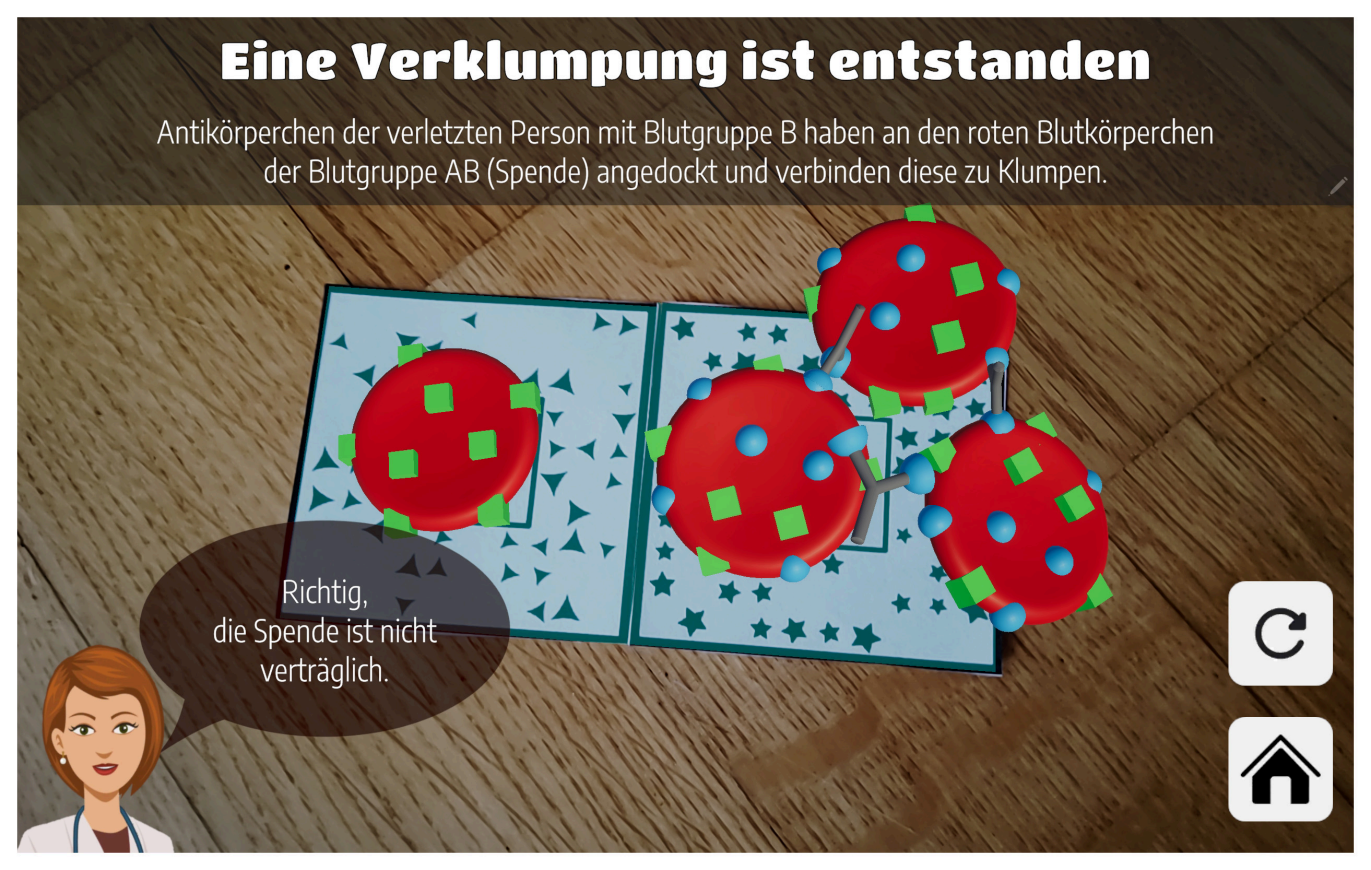	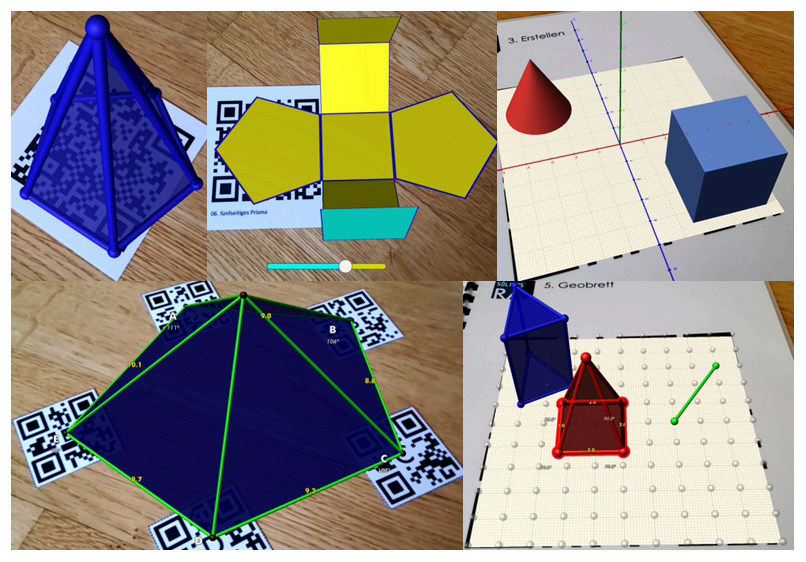
(Schmid et al., 2018)	(idea by Vivien Wilzbach, dev. in collab. w/HSLU)	(Amorim and de Oliveira Freitas, 2023)
AR paper circuits	Insight Heart	SPATIAL AR
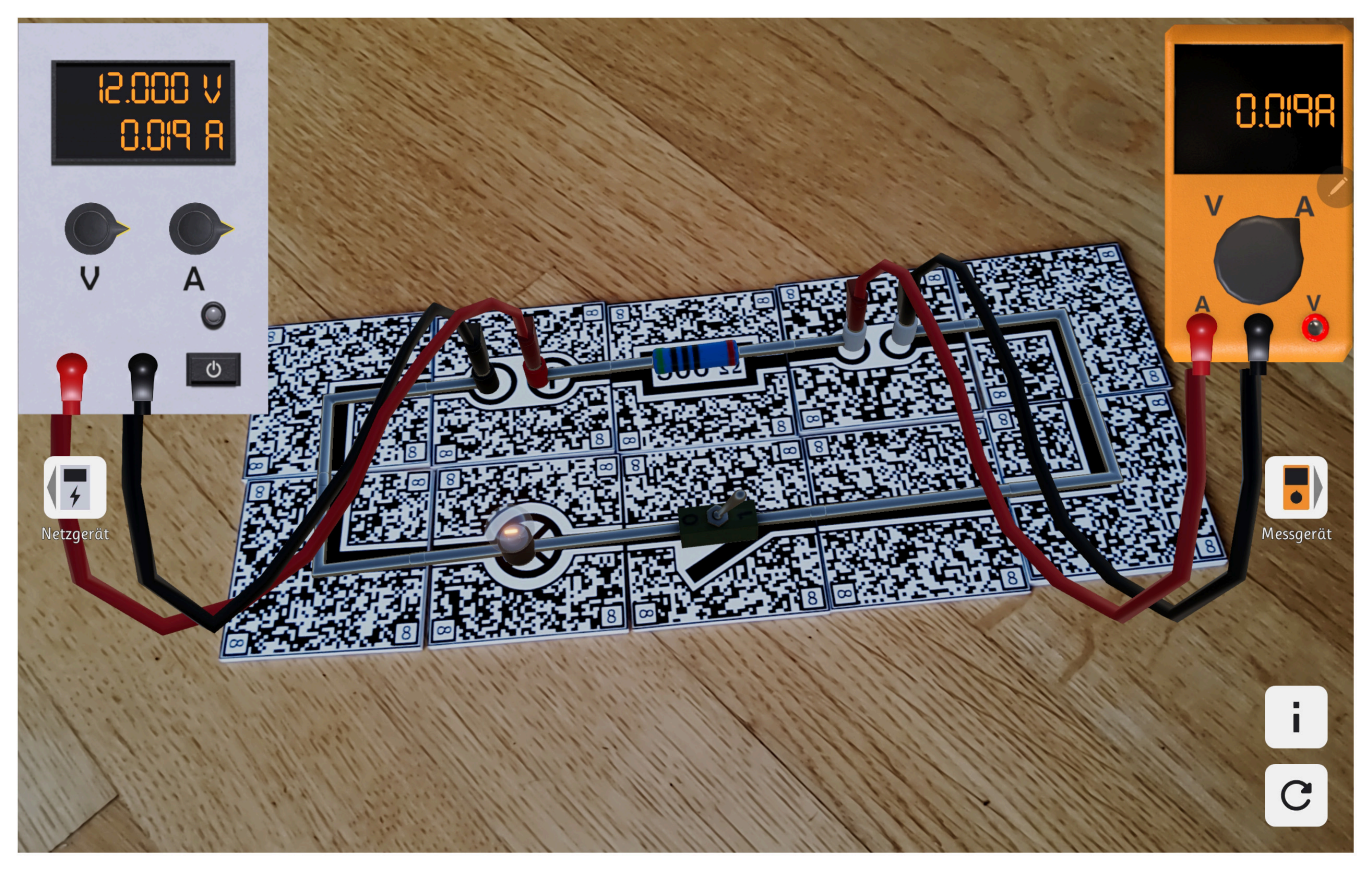	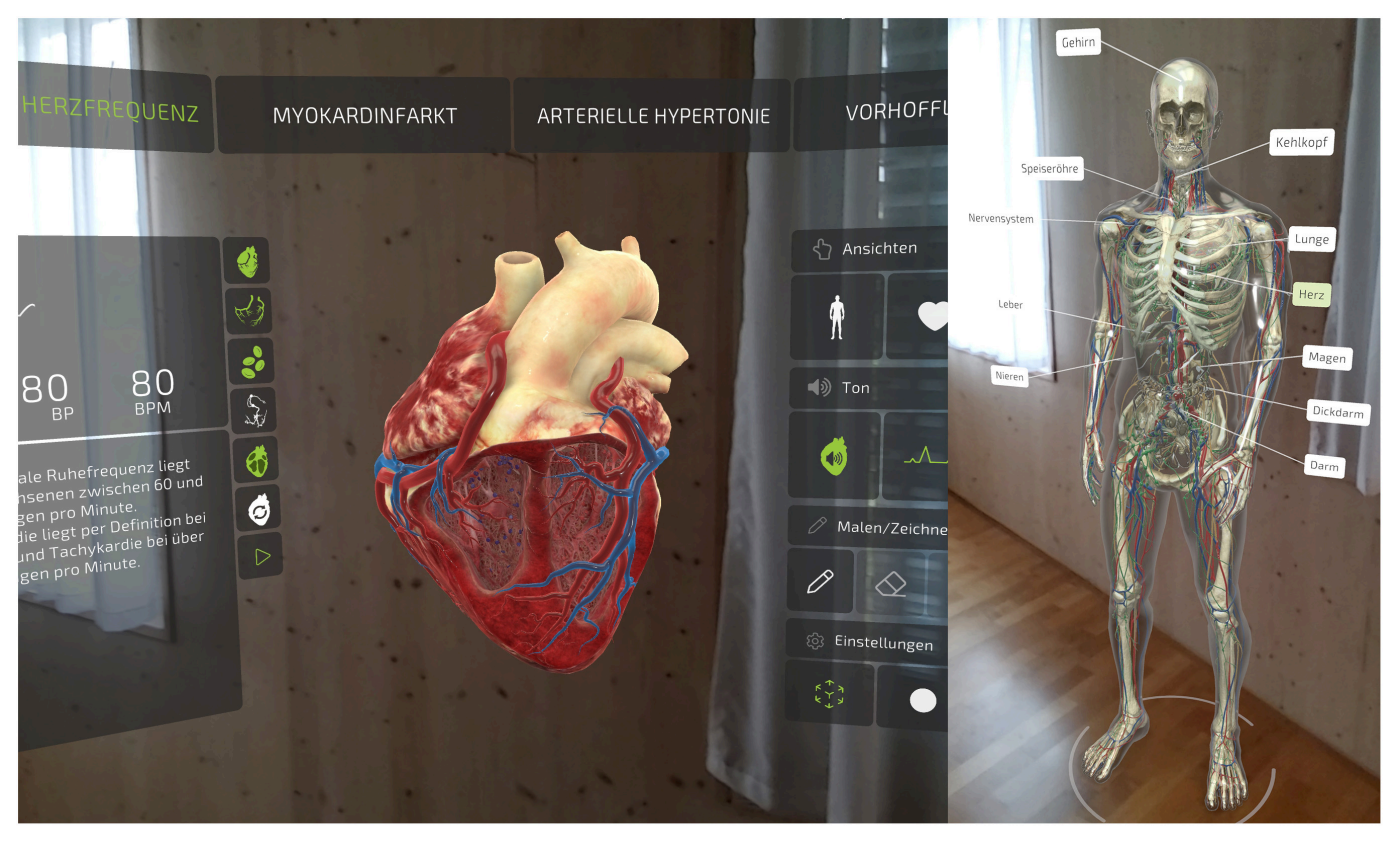	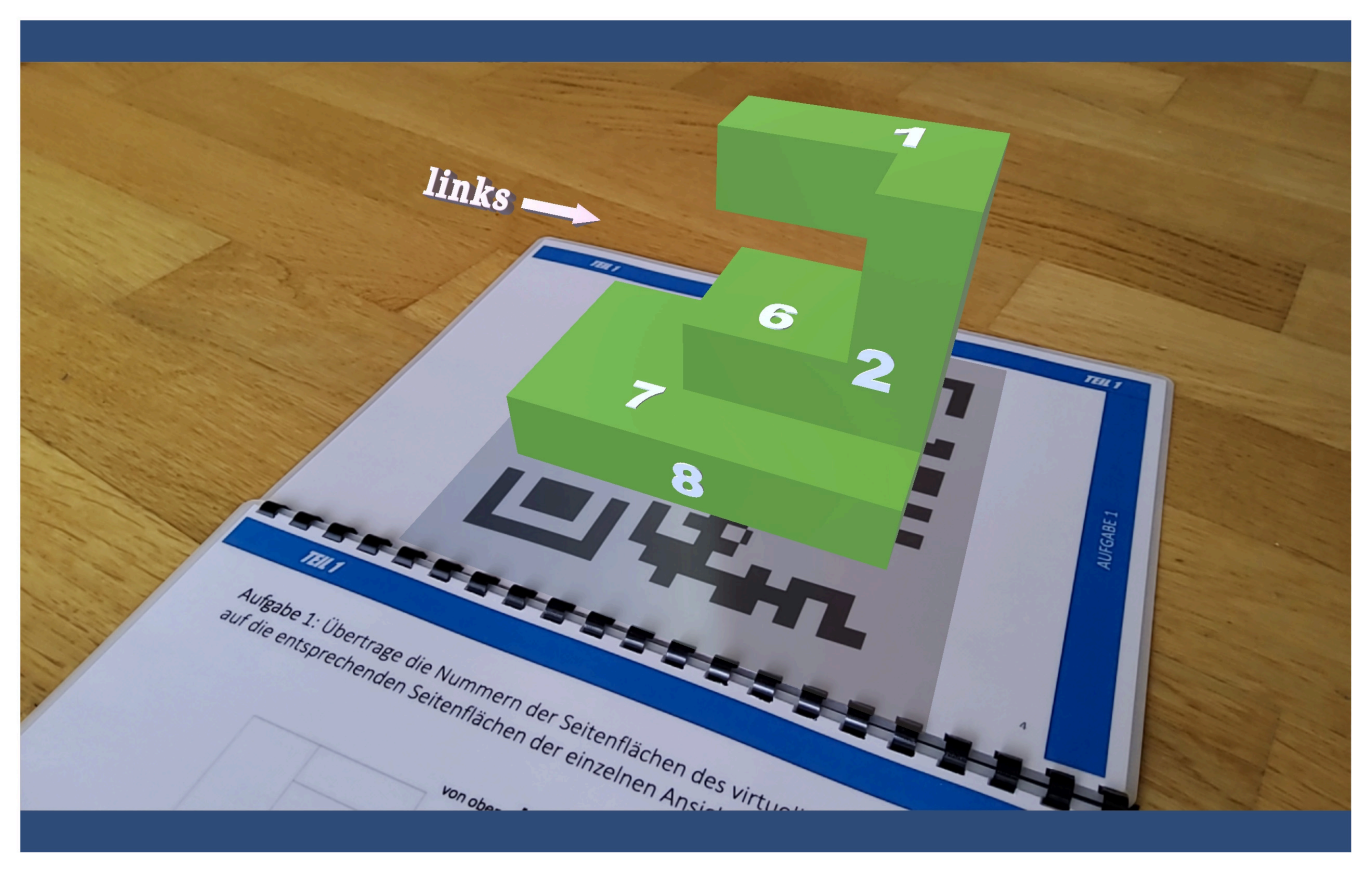
(dev. in collab. w/HSLU)	(ANIMA RES, 2022)	(Özçakir and Çakiroglu, 2021a,b)

Screenshots taken by the authors, reproduced with the consent of the application's developers.

After evaluating and selecting augmented reality applications and planning teaching units with them, the prospective teachers answered further questions. These included completing self-assessments of PCK, TPK and TPACK, based on a validated questionnaire by Schmid et al. (2020a). Each scale consists of four items, which are answered using a five-point Likert scale. Testing and evaluating the AR applications took around 60 min in total. Including the introduction to the study and the final questions, the total survey time for mathematics and biology was around 90 min. The physics survey took around 100 min as it included an excerpt from a PCK test on electric circuits (Schödl, 2018).

### 2.2 Sample

#### 2.2.1 Data collection

The survey was conducted in STEM education modules at the Universities of Teacher Education in Lucerne, Zurich, Bern and Thurgau. Module conditions varied, but in most cases, participation in the study was recognized as course attendance and could be compensated through alternative means. As an incentive, students received access to the tested AR applications as well as a curated list of free AR applications for classroom use. Although only a small number of students chose not to participate, the exact number is unknown due to variability in module formats. Nevertheless, there is no indication of systematic self-selection bias. Because of the time needed to test each application, participants could only assess two applications per session. Since data collection was conducted within modules, all students in each module group evaluated the same two AR applications. With few exceptions, these applications were thematically aligned with the content of the respective modules. The assignment of application pairs to the module groups was based on a randomized yet balanced design, ensuring comparable group sizes across subjects.

#### 2.2.2 Participants

A total of *N* = 305 prospective lower secondary school teachers, who are being trained to teach STEM subjects to 12-15-year-old pupils, participated in the study. The participants had an average age of 24.45 years (*SD* = 5.33). Of the participants, 52.5% were female and 47.5% were male. Table 2 illustrates the distribution of teaching subject choices within STEM disciplines. In Swiss teacher education programs, it is common for prospective teachers to choose more than one subject to teach. The most frequently selected STEM subjects were Science and Technology and Mathematics, followed by Media and Computer Science. Further explanations of the individual subjects can be found in the note below Table 2.

**Table 2:** Distribution of teaching subjects chosen by participants.

**Teaching subject choice**	**Number of participants**	**Percentage of participants**
Mathematics	234	76.7%
Science and technology (including biology, chemistry, and physics)^a^	235	77.0%
Media and computer science^b^	110	36.1%
STEM elective (science, technology, engineering, and mathematics)^c^	25	8.2%

*N* = 305. In Switzerland, prospective teachers typically choose multiple subjects; therefore, percentages sum to more than 100%.

^a^The Swiss school subject Science and Technology encompasses the traditional natural sciences—biology, chemistry, and physics—as well as an interdisciplinary component that includes, among other things, an emphasis on technical aspects.

^b^Media and Computer Science is a compulsory subject in Swiss schools that integrates media education and basic computer science skills as part of the curriculum.

^c^The STEM elective is an integrated compulsory elective subject in Lucerne encompassing multiple disciplines (science, technology, engineering, and mathematics).

*n* = 45 participants took part in the study twice and *n* = 11 participants took part three times. This resulted in the following subsamples per subject: mathematics (*n* = 123), biology (*n* = 120) and physics (*n* = 129). To investigate how augmented reality applications are evaluated and selected during teacher education, the prospective teachers were divided into two groups for data analysis: undergraduate students (those in their bachelor's program) and graduate students (those in their master's program). Table 3 provides an overview of the subsamples of this study.

**Table 3:** Overview of the sub-samples of this study.

**Level of education**	**Mathematics**	**Biology**	**Physics**
Undergraduate students	81	88	83
Graduate students	42	32	46
Total	123	120	129

*N* = 305 people carried out n = 372 AR evaluations.

### 2.3 Data analysis

#### 2.3.1 Post hoc sensitivity analyses

To assess the statistical power of the analyses conducted in this study, *post hoc* sensitivity analyses assuming α = 0.05 and power (1–β) = 0.80 were performed using G^*^Power (Faul et al., 2009). Table 4 presents an overview of these sensitivity results by subject and test. For the Mann-Whitney U tests comparing undergraduate and graduate students within each STEM discipline, the minimum detectable effect sizes (Cohen's *d*) ranged from 0.54 to 0.60. Accordingly, for the analysis addressing research questions 1 and 3, the study had 80% power to detect effects of *d* ≥ 0.60; however, it may have lacked sufficient power to reliably detect smaller effects, including the conventional medium effect size of *d* = 0.50, which was below the detection threshold. For the Pearson chi-squared tests examining group differences in the choice of AR applications (research question 2), the minimum detectable effect sizes (Cramér's *V*) ranged from 0.25 to 0.30, depending on the subject and the number of response options (two under ideal, four under real-world conditions). Thus, the study was powered to detect moderate effects (*V* ≥ 0.30) but may not have been sensitive enough to detect small effects.

**Table 4:** Post hoc sensitivity analyses.

**Subject**	***N***	***n* (UG)**	***n* (G)**	**MWU: min. detectable Cohen's *d***	***χ^2^*(*1*): min. detectable Cramér's *V***	***χ^2^*(*3*): min. detectable Cramér's *V***
Mathematics	123	81	42	0.55	0.25	0.30
Biology	117	85	32	0.60	0.26	0.30
Physics	127	82	45	0.54	0.25	0.29

Assuming a significance level (α) of 0.05 and a statistical power (1–β) of 0.80. UG = undergraduate students; G = graduate students; MWU = Mann-Whitney U test; χ^2^(1) and χ^2^(3) = Pearson chi-square tests with degrees of freedom 1 and 3. To account for the five missing cases related to research question 3, the reported values for N and n have been adjusted accordingly.

#### 2.3.2 Qualitative content analysis

The text responses of the prospective teachers were analyzed using a qualitative content analysis following the deductive-inductive approach outlined by Kuckartz and Rädiker (2022). The main categories were derived from the knowledge areas of the TPACK model (see Section 1.2; Koehler and Mishra, 2009). Building on the conceptual synthesis by Petko et al. (2025), contextual knowledge (XK) was added as a distinct knowledge domain, resulting in a total of eight main categories. The subcategories were derived from the research literature of each knowledge area and the participants' responses. The development of the category system is described in detail in another article (Küng et al., submitted). An abridged, translated version of the original 30-page codebook can be found in the Supplementary material. The following participant statement is provided to illustrate the application of the category system: “As with any model, you have to address the fact that this is a mental model and does not represent reality (electricity does not fall from a higher point to a lower point—waterfall model).” (122; pos. 13, translated) This person bases their argument on their pedagogical content knowledge (PCK), specifically their model understanding. Therefore, the PCK subcategory “7.3.1.2 Model understanding” was coded (see Supplementary material for a description of this category).

To assess intercoder reliability, the Kappa coefficient (κn) as proposed by Brennan and Prediger (1981) was calculated using MAXQDA for each subject based on ten cases. The resulting agreement values at both the main and subcategory levels are presented in Table 5. According to Wirtz and Casper (2002), these values are marginal or not fully satisfactory. Despite the use of a comprehensive codebook, clearly defined coding guidelines, and intensive coder training, the level of agreement could not be further improved. A key challenge was the large number of categories, which increased the complexity of the coding process. Nevertheless, the detailed categorization system was necessary to adequately address the research questions and contribute meaningfully to the research discourse. To maximize reliability during the distributed coding of the remaining cases, text segments with ambiguous interpretations were coded through consensus.

**Table 5:** Intercoder reliability for subjects by main and subcategories.

**Category level**	**Mathematics**	**Biology**	**Physics**
Main categories	κ_n_ = 0.63	κ_n_ = 0.68	κ_n_ = 0.73
Subcategories	κ_n_ = 0.63	κ_n_ = 0.65	κ_n_ = 0.67

κ_*n*_ = Kappa coefficient as proposed by (Brennan and Prediger 1981) based on ten cases per subject.

#### 2.3.3 Quantitative analyses of coded categories, application choices, and self-assessments

Following the coding process, quantitative analyses were conducted based on the frequency with which the TPACK knowledge areas and their subtopics were referred to by the prospective teachers. This enables conclusions about which areas of knowledge are currently in focus in teacher training, and which should be strengthened further. Mann-Whitney U tests were performed for each subject to determine whether the focus on the TPACK knowledge areas and their subtopics differed between undergraduate and graduate students. Pearson chi-squared tests were performed for each subject to determine whether undergraduate and graduate students selected different augmented reality applications under ideal and under real-world conditions.

Before analyzing group differences based on the TPACK self-assessment questionnaire excerpt from Schmid et al. (2020a), the internal consistencies of the PCK, TPK and TPACK scales were evaluated using Cronbach's alpha (see Table 6). While the internal consistency of the PCK and TPACK scales were satisfactory, the consistency of the TPK scale was only borderline acceptable, which is further addressed in Section 6. Mean values were calculated for each scale. These values were then used in Mann-Whitney U tests to examine whether there are differences in the TPACK, PCK and TPK self-assessment between undergraduate and graduate students. These tests were carried out once overall and once separately by subject.

**Table 6:** Cronbach's alpha for the PCK, TPK and TPACK self-assessment scales according to Schmid et al. (2020a).

**Self-assessment scale**	**Mathematics (*n* = 123)**	**Biology (*n* = 120)**	**Physics (*n* = 129)**
PCK	0.81	0.88	0.81
TPK	0.68	0.70	0.69
TPACK	0.86	0.84	0.74

## 3 Results

### 3.1 RQ1—Differences in TPACK references between undergraduate and graduate students

Mann-Whitney U tests were performed for each subject to evaluate whether the code frequencies of the TPACK knowledge areas and their subtopics varied according to current educational level (undergraduate/graduate). The results are shown in Table 7. Two differences between undergraduate and graduate students were reflected in two subject evaluations. In both the assessments of the mathematics and the physics applications, graduate students argued more frequently with technological content knowledge (TCK) (especially knowledge about the possibilities of technologies in relation to subject knowledge) than undergraduate students did. Undergraduate students, on the other hand, argued more frequently with technological pedagogical knowledge (TPK) (especially knowledge about the use of media in teaching and media psychology) in these two subjects. The other differences in code frequencies between undergraduate and graduate students only occurred in one subject evaluation each. In the assessment of the mathematics applications, the graduate students argued more frequently with technological knowledge (TK) (especially application knowledge), than undergraduates did. In the assessment of the biology applications, graduate students argued more frequently with the requirement level, a subcategory of pedagogical content knowledge (PCK). In the assessment of the physics applications, graduate students argued more frequently with facts, i.e. verbalizable knowledge, a subcategory of content knowledge (CK), as well as more with pedagogical content knowledge (PCK) (especially knowledge about making content understandable, including model competence, more precisely model knowledge and use, as well as general visualization cues) and with more knowledge about making content understandable using the medium, a subcategory of TPACK. The effect sizes, as measured by Cohen's *d* indicate small to large effects (see Table 7). The differences in model competence and its subcategory model knowledge and use show the largest effect sizes.

**Table 7:** Differences in code frequencies between undergraduate and graduate students by subject evaluation (Mann-Whitney U tests).

**Subject and subsamples**	**Category (No./Name)**	***Mdn* (UG)**	***M* (UG)**	***SD* (UG)**	***Mdn* (G)**	***M* (G)**	***SD* (G)**	***z***	***p***	***d***
Mathematics (*N* = 123; *n* UG = 81; *n* G = 42)	2. Technological knowledge (TK)	2.00	2.64	2.43	4.00	3.95	2.73	−2.75	0.006	−0.51
	2.1. Application knowledge	2.00	2.64	2.43	4.00	3.95	2.73	−2.75	0.006	−0.51
	3. Technological content knowledge (TCK)	1.00	0.74	0.89	1.00	1.83	1.91	−3.07	0.002	−0.58
	3.2. Knowledge about the possibilities of technologies in relation to subject knowledge	1.00	0.74	0.89	1.00	1.83	1.91	−3.07	0.002	−0.58
	4. Technological pedagogical knowledge (TPK)	4.00	4.22	2.44	3.00	3.21	1.96	2.71	0.007	0.50
	4.1. Knowledge about the use of media in teaching and media psychology	4.00	4.17	2.33	3.00	3.21	1.96	2.68	0.007	0.50
Biology (*N* = 120; *n* UG = 88; *n* G = 32)	7.1.1. Requirement level	0.00	0.30	0.66	0.50	0.72	0.89	−3.05	0.002	−0.58
Physics (*N* = 129; *n* UG = 83; *n* G = 46)	3. Technological content knowledge (TCK)	0.00	1.07	1.60	1.00	1.87	1.59	−3.50	< 0.001	−0.65
	3.2. Knowledge about the possibilities of technologies in relation to subject knowledge	0.00	1.06	1.60	1.00	1.87	1.59	−3.57	< 0.001	−0.66
	4. Technological pedagogical knowledge (TPK)	2.00	2.08	2.09	1.00	1.33	1.71	2.37	0.018	0.43
	4.1. Knowledge about the use of media in teaching and media psychology	2.00	2.02	2.05	1.00	1.33	1.71	2.30	0.022	0.41
	6.1. Facts: Verbalizable knowledge	0.00	0.08	0.32	0.00	0.28	0.69	−2.37	0.018	−0.43
	7. Pedagogical content knowledge (PCK)	3.00	3.93	2.92	5.00	5.00	3.00	−2.14	0.032	−0.38
	7.3. Knowledge about making content understandable	2.00	2.76	2.31	4.00	3.87	2.13	−3.02	0.003	−0.55
	7.3.1. Model competence	0.00	0.40	0.91	1.00	1.15	1.30	−4.33	< 0.001	−0.82
	7.3.1.1. Model knowledge and use	0.00	0.37	0.85	1.00	1.15	1.30	−4.45	< 0.001	−0.85
	7.3.2.3. General visualization cues	0.00	0.57	0.83	1.00	0.93	0.95	−2.33	0.020	−0.42
	8.3. Knowledge about making content understandable using the medium	1.00	0.72	0.75	1.00	1.09	0.81	−2.51	0.012	−0.45

UG, undergraduate students; G, graduate students. Only statistically significant results (*p* < 0.05) are shown.

### 3.2 RQ2—Differences in the selection of AR applications between undergraduate and graduate students

Pearson chi-squared tests show that the current level of education (undergraduate/graduate) is related to the selection of the augmented reality application under ideal conditions in the assessment of the mathematics (*X*^2^ (1, *N* = 123) = 4.7, *p* = 0.030) and the biology (*X*^2^ (1*, N* = 120) = 8.2, *p* = 0.004) but not the physics (*X*^2^ (1, *N* = 129) = 1.6, *p* = 0.211) applications. However, the relationship is not strong (Cramér's *V* < 0.30) for either the assessment of the mathematics (Cramér's *V* = 0.195) or biology (Cramér's *V* = 0.262) applications. In the assessment of the mathematics applications under ideal conditions, more undergraduate students chose *Sólidos RA* (74.1%) than *SPATIAL AR* (25.9%), while the ratio is more balanced among graduate students (54.8% *Sólidos RA*, 45.2% *SPATIAL AR*). In the case of the assessment of the biology applications under ideal conditions, more undergraduate students chose *Insight Heart* (84.1%) than *Blood group compatibility* (15.9%), whereas the ratio is more balanced among graduate students (59.4% *Insight Heart*, 40.6% *Blood group compatibility*).

When selecting under real-world conditions (everyday teaching context), the only significant difference between undergraduate and graduate students occurred in the assessment of the biology applications (*X*^2^ (3, *N* = 120) = 9.3, *p* = 0.026), and the relationship is not strong (Cramér's *V* = 0.278). Similarly to the ideal conditions, undergraduate students chose *Insight Heart* more often, while graduate students made a more balanced choice, with a slight preference for *Blood group compatibility*. As shown in Table 8, undergraduate students also chose both applications more often than graduate students.

**Table 8:** Distribution of selected augmented reality applications in biology (%), by student level (undergraduate/graduate).

**Student level**	**Blood group compatibility**	**Insight Heart**	**Both applications**	**None of the applications**
Undergraduate students	10.2%	26.1%	52.3%	11.4%
Graduate students	31.3%	21.9%	31.3%	15.6%

### 3.3 RQ3—Differences in self-assessed PCK, TPK, and TPACK between undergraduate and graduate students

To provide a comprehensive overview of prospective teachers' self-assessed competencies in PCK, TPK, and TPACK, data from undergraduate and graduate students were initially analyzed collectively (first participation; *N* = 300; *n* undergraduates = 197; *n* graduates = 103; *n* missing = 5). Participants rated their perceived competencies on a five-point Likert scale (1 = strongly disagree, 5 = strongly agree). The results were as follows: PCK (*Mdn* = 4.00, *M* = 3.81, *SD* = 0.62), TPK (*Mdn* = 3.88, *M* = 3.83, *SD* = 0.55), and TPACK (*Mdn* = 3.75, *M* = 3.68, *SD* = 0.62). Overall, the prospective teachers perceived their competencies as moderate. Among the three knowledge domains, TPACK received slightly lower ratings compared to PCK and TPK.

To examine potential differences in the self-assessment of PCK, TPK and TPACK between undergraduate and graduate students, the participants' initial study participation was analyzed, regardless of the subject evaluation (*N* = 300; *n* undergraduates = 197; *n* graduates = 103; *n* missing = 5; see Table 9). Although no significant differences were identified in the self-assessment of TPK (*z* = −1.21, *p* = 0.227) or TPACK (*z* = −0.65, *p* = 0.519), a significant difference was found in the self-assessment of PCK (*z* = −3.15, *p* = 0.002). According to Cohen (1988), the effect size of *d* = −0.37 corresponds to a small effect. Graduate students (*Mdn* = 4.00, *M* = 3.96, *SD* = 0.59) rate their PCK competencies significantly higher than undergraduate students (*Mdn* = 3.75, *M* = 3.74, *SD* = 0.62). A value of 4 on the five-point Likert scale indicates 'Tend to agree', the second highest level.

**Table 9:** Differences in self-assessed PCK, TPK, and TPACK between undergraduate and graduate students, by subject (Mann-Whitney U tests).

**TPACK area**	**Subject**	***Mdn* (UG)**	***M* (UG)**	***SD* (UG)**	***Mdn* (G)**	***M* (G)**	***SD* (G)**	***z***	***p***	***d***
PCK	All subjects	3.75	3.74	0.62	4.00	3.96	0.59	−3.15	0.002	−0.37
	Mathematics	4.00	3.85	0.62	4.00	4.01	0.47	−1.56	0.118	−0.28
	Biology	4.00	3.80	0.62	4.00	4.06	0.73	−2.36	0.018	−0.45
	Physics	3.63	3.57	0.58	3.75	3.81	0.51	−1.79	0.074	−0.32
TPK	All subjects	4.00	3.85	0.56	3.75	3.79	0.53	−1.21	0.227	−0.14
	Mathematics	4.00	3.87	0.52	4.00	3.80	0.56	−0.51	0.614	−0.09
	Biology	4.00	3.89	0.61	4.00	3.92	0.41	−0.13	0.899	−0.02
	Physics	3.75	3.74	0.52	3.75	3.77	0.57	−0.11	0.912	−0.02
TPACK	All subjects	3.75	3.66	0.66	3.75	3.72	0.52	−0.65	0.519	−0.07
	Mathematics	3.75	3.69	0.75	4.00	3.79	0.47	−0.84	0.400	−0.15
	Biology	3.75	3.67	0.69	3.75	3.65	0.57	−0.29	0.773	−0.05
	Physics	3.75	3.66	0.54	3.75	3.74	0.56	−0.55	0.581	−0.10

UG, undergraduate students; G, graduate students. All subjects (*N* = 300; *n* UG = 197; *n* G = 103; *n* missing = 5); Mathematics (*N* = 123; *n* UG = 81; *n* G = 42); Biology (*N* = 117; *n* UG = 85; *n* G = 32; *n* missing = 3); Physics (*N* = 127; *n* UG = 82; *n* G = 45; *n* missing = 2).

When examining differences in self-assessed PCK, TPK and TPACK per subject evaluation, only one significant difference was found between undergraduate and graduate students: in biology, relating to PCK (*z* = −2.36, *p* = 0.018). According to Cohen (1988), this difference also indicates a small effect size (*d* = −0.45). As observed across all subjects, in the biology evaluation graduate students (*Mdn* = 4.00, *M* = 4.06, *SD* = 0.73) rated their PCK higher than undergraduate students did (*Mdn* = 4.00, *M* = 3.80, *SD* = 0.62). The remaining Mann-Whitney U-tests were not significant (see Table 9).

## 4 Discussion

This study examined three research questions focusing on differences between undergraduate and graduate students in lower secondary STEM education. RQ1 investigated how students referred to TPACK areas when evaluating AR applications. In the mathematics and physics assessment, graduate students more often drew on TCK, while undergraduates emphasized TPK. Distinct subject-assessment-specific patterns were observed, with the strongest differences between undergraduate and graduate students in the physics assessment, particularly regarding the PCK subcategory model knowledge and use. RQ2 addressed students' selection of AR applications. Under ideal conditions, undergraduates tended to prefer specific applications more strongly, while graduates distributed their choices more evenly; under real-world conditions, this difference was only evident in the biology assessment. RQ3 explored self-assessments of PCK, TPK, and TPACK. Graduate students rated their PCK significantly higher than undergraduates (across all subject assessments and in the biology assessment) while no significant differences were found in self-assessed TPK or TPACK.

### 4.1 RQ1—Differences in TPACK references between undergraduate and graduate students

References to TPACK knowledge areas and their subdomains vary between undergraduate and graduate students. However, development in the integrative domain of TPACK throughout teacher education was observed only in the assessment of physics applications and was limited to a single sub-dimension (8.3. Knowledge about making content understandable using the medium) with a small effect size (*p* = 0.012, *d* = −0.45). While this may indicate that a more consistent emphasis on fostering TPACK during teacher training could be beneficial, limited statistical power—particularly in smaller subgroups (see Section 2.3.1)—may have hindered the detection of more subtle effects.

In both the assessments of the mathematics and the physics applications, graduate students refer more frequently to technological content knowledge (TCK), particularly regarding the possibilities of technologies in relation to subject knowledge. This suggests that TCK is fostered during training for teaching mathematics and physics. However, it is not possible to determine whether this occurs directly or through the development of related knowledge components, namely technological knowledge (TK) and content knowledge (CK). Nevertheless, the finding that graduate students refer more often to TK in the assessment of the mathematics applications and to a subdimension of CK in the assessment of the physics applications suggests that TCK may be indirectly supported through the advancement of these foundational domains. While graduate students refer more often to TCK, undergraduate students refer more often to TPK in the assessments of the mathematics and the physics applications. This could suggest that TPK decreases through teacher training. However, it should be noted that discussions about TPK primarily focused on the use of media in teaching and media psychology. More specifically, the prospective teachers focused on the motivation to learn through technology and distractions that technical devices can cause. Undergraduates may focus more on these topics because they have less teaching experience and are therefore more uncertain about using technology in the classroom.

The finding that in the assessment of the physics applications graduate students refer to pedagogical content knowledge (PCK) more frequently than undergraduates do, suggests that teacher training successfully promotes PCK regarding the topic of electrical circuits. Specifically, graduate students refer more often to their knowledge about making content understandable, including model competence (especially model knowledge and use) and general visualization cues. These aspects are particularly relevant in physics education, where visual models are often necessary to support an understanding of abstract phenomena (e.g. Winkelmann et al., 2022). Differences in PCK also emerge in the assessment of the biology applications, where graduate students more frequently refer to the requirement level of a task. This involves adapting content to students' prior knowledge and learning needs. This finding suggests that graduate students are better able to assess the suitability of learning materials for specific learner groups due to their training and experience.

### 4.2 RQ2—Differences in the selection of AR applications between undergraduate and graduate students

The findings suggest that the selection of augmented reality applications varies depending on the level of teacher education. Under ideal conditions, undergraduate students show a clearer preference for a specific application in the assessments of the mathematics and the biology applications, whereas graduate students tend to distribute their choices more evenly between the two options. In both subject assessments, undergraduates tend to favor the application offering a broader range of technological features, even if it is less clearly tailored to pedagogical use. This pattern may indicate that undergraduate students are more influenced by an application's technological novelty or complexity, whereas graduate students appear to make more deliberate, goal-oriented decisions, potentially drawing on the professional knowledge they have developed during their studies.

When considering application choices under real-world conditions (in everyday teaching context), an additional trend emerges. In the assessment of the biology applications, undergraduate students are more likely than graduate students to report that they would use both applications. This may reflect a lower level of critical appraisal among undergraduates when it comes to evaluating the suitability of AR applications for STEM teaching. In contrast, graduate students may already possess clearer criteria for assessing the relevance and classroom applicability of digital learning resources, making them more selective in their use.

### 4.3 RQ3—Differences in self-assessed PCK, TPK, and TPACK between undergraduate and graduate students

Consistent with the findings of the German study by Zinn et al. (2022), the prospective teachers in the present study rated their competencies in the technology-related domains (in this case, TPK and TPACK) as moderate. This suggests that prospective teachers possess a moderate level of confidence in their TPK and TPACK. In the present study, prospective teachers rated their PCK as highly as their TPK and TPACK. A further parallel to Zinn et al. (2022) is the significant improvement observed in self-assessed PCK, while no statistically significant gains were found in the technology-related domains TPK and TPACK. This pattern is also reflected in the German study by Weidlich and Kalz (2023). The finding that prospective teachers rate their PCK more highly toward the end of their training suggests an increase in confidence in this domain over the course of their education. In contrast, the absence of a similar increase in self-assessed TPK and TPACK may indicate that teacher education does not effectively enhance confidence in these areas. Alternatively, as graduate students engaged more deeply with educational content and authentic teaching experiences than undergraduate students, they may have developed a more accurate and nuanced self-assessment of their technology-related competencies. However, it is also possible that limited statistical power (see Section 2.3.1)—particularly in smaller subgroups—hindered the detection of more subtle differences in self-assessed TPK and TPACK.

The increase in self-assessed competencies across all TPACK domains over teacher training reported in the Finnish study by Valtonen et al. (2019) could not be replicated under Swiss conditions. While Valtonen et al. (2019) observed the greatest improvements in PCK, PK, and TPACK self-assessment over teacher training, the present study found significant improvements only in PCK; PK was not assessed. Similarly, the Chinese study by Li et al. (2022) reported increases in TPK and TPACK but no significant improvement in PCK- contrary to the findings of the present study, in which of the three considered domains (TPK, TPACK and PCK) only PCK self-assessment showed a statistically significant increase over teacher training. Overall, these international comparisons indicate that Switzerland's situation is closer to that in Germany than in Finland or China. One possible explanation lies in the geographical and cultural proximity to Germany and a shared academic tradition, particularly with respect to the emphasis on pedagogical content knowledge (PCK) in teacher education and educational research (e.g. Werler and Tahirsylaj, 2020).

## 5 Conclusion and implications

This study shows that the knowledge-based evaluation and selection of augmented reality (AR) applications, as well as self-assessed pedagogical content knowledge (PCK), change over the course of teacher training. However, there remains room for improvement in prospective teachers' digital competencies. A more pronounced reference to TPACK was only observed in the assessment of the physics applications, with graduate students referring to one subcategory of TPACK more frequently than undergraduates. This suggests that the integration of TPACK could be promoted more systematically across subjects and stages of teacher education. However, it should be noted that limited statistical power- particularly in smaller subgroups (see Section 2.3.1)- may have hindered the detection of more subtle developments of TPACK over the course of teacher training. When selecting AR applications, graduate students demonstrated greater differentiation between the two AR applications in the assessments of the biology and the mathematics applications. Nevertheless, they could be encouraged to demonstrate an increasingly strong preference for the option that is more tailored for educational use over the technologically advanced option. Similarly, self-assessments of PCK, TPK and TPACK revealed a significant increase only in PCK; no substantial increases in self-assessed TPK or TPACK were observed throughout the course of teacher training. However, it is possible that limited statistical power—particularly in smaller subgroups (see Section 2.3.1)- may have prevented the detection of more subtle developments in these areas. In conclusion, this study corroborates the findings of previous studies (Senkbeil et al., 2021; Johnson et al., 2023; see Section 1.4) that prospective teachers' digital competencies could be further developed. According to the Will, Skill, Tool (WST) model, teachers' digital competencies (i.e. Skill) are essential for the effective integration of digital learning resources into classroom practice (Knezek and Christensen, 2016; see Section 1.1). Similarly, the TPACK framework (Koehler and Mishra, 2009; see Section 1.2) emphasizes the importance of combining technological, pedagogical and content knowledge to enable teachers to use technology effectively in subject-specific contexts.

To further strengthen the digital competencies of prospective teachers, teacher education could adopt a more systematic and integrated approach to developing them, such as drawing on the SQD model proposed by Tondeur et al. (2012) (see Section 1.3). Teacher educators should serve as role models, provide practical examples, and facilitate hands-on experimentation with educational technologies. Regarding the TPACK model (Koehler and Mishra, 2009; see Section 1.2), it is important to integrate such training into subject-specific courses. Although meta-analyses have confirmed the positive effects of interventions targeting digital competence development (Ning et al., 2022; Fabian et al., 2024), these interventions must still be systematically embedded in teacher education programs. Several studies have shown that many teacher educators lack sufficient digital competencies themselves and that digital learning resources are not yet adequately integrated into teacher training curricula (Tondeur et al., 2019; Voithofer and Nelson, 2020; Lindfors et al., 2021).

In this study, augmented reality applications were used as representative examples of digital learning resources. Existing research suggests that AR seems to play only a minor role in teacher education (Schwaiger et al., 2024; Trust et al., 2021; Avila-Garzon et al., 2021), and that teachers often lack the necessary competencies to use this technology effectively (Belda-Medina and Calvo-Ferrer, 2022; Freese et al., 2023). The present findings align with these observations.

## 6 Limitations and research outlook

The present study focused on augmented reality (AR) applications in three STEM subjects to evaluate prospective teachers' digital competencies in a subject- and technology-specific manner. Consequently, the generalizability of the findings to other technologies (e.g. explainer videos) or school subjects (e.g. languages) is inherently limited. The results may differ, particularly when it comes to technologies with which prospective teachers are already familiar, in contrast to the largely unfamiliar technology of AR (see section 1.5). Furthermore, the study was conducted within the context of Swiss teacher education. As discussed in section 4.3, the findings may differ in other national contexts, or even between Swiss institutions, due to variations in structure and curriculum. Future research is recommended to replicate and expand upon the current study design to explore digital competencies in a range of different contexts.

A methodological limitation relates to sample size and statistical power. Although the study had 80% power to detect effects of *d* ≥ 0.60 for the Mann-Whitney U tests and *V* ≥ 0.30 for the Pearson chi-squared tests, the power to identify smaller effects, particularly in the graduate biology subgroup, was limited. Consequently, non-significant results should be interpreted with caution. The relatively high minimum detectable effect sizes result partly from the limited overall sample size and partly from the uneven distribution between undergraduate and graduate students. Such imbalances are common in higher education field studies, as undergraduate programs usually have a longer duration than graduate programs, leading to a larger number of enrolled undergraduates at any given time. Future research should aim to achieve larger and more balanced sample sizes, particularly within groups where power was limited (e.g., graduate biology), to improve sensitivity to smaller effects. Additionally, longitudinal designs could provide valuable insights into developmental trajectories across educational stages, complementing the study's cross-sectional approach.

The intercoder reliability was marginal (Wirtz and Casper, 2002), despite the use of a detailed codebook, clear guidelines, and coder training. A main challenge was the large number of categories, which added complexity but was necessary to address the research questions. To ensure consistency, ambiguous cases were coded by consensus. This limitation is discussed in greater detail in Küng et al. (submitted). Future studies might reduce category complexity or use interviews to support clearer interpretation.

In the survey instrument for the self-assessment, the internal consistency of the TPK subscale was slightly below the desired threshold (Cronbach's α = 0.68–0.70). Although the present study yielded a higher reliability for PCK compared to Schmid et al. (2020a; α = 0.79), their reported reliability for TPK (α = 0.81) and TPACK (α = 0.87) exceeded those observed in the current study. This discrepancy may be attributable to the specific study context: participants completed the TPACK self-assessment after testing AR applications, whereas the items referred more generally to digital technologies. Despite the general answering instructions, the specific context of AR may have led to uncertainty about how to interpret the subsequent items. To ensure comparability with prior research, the study adhered to the established and previously validated scale. Further validation of the scale within the current sample was beyond the scope of this study. Future studies with larger samples should further investigate the reliability and validity of this instrument in diverse settings.

With respect to the analysis, the findings for research question 1 are based on the frequency of coded categories, representing the relative knowledge-based focus of prospective teachers when evaluating AR applications. However, this approach only captures the quantity, not the quality, of their reasoning. As the accuracy and depth of the participants' arguments were not evaluated, there is clearly a need for additional qualitative analysis. Subsequent analysis of this study will also explore the additional factors that lead to higher levels of TPACK. Incorporating variables such as attitudes toward technology will provide a more nuanced understanding of the development of digital competence.

This study went beyond traditional self-assessment by employing methods aligned with vignette studies to capture digital competencies. It focused specifically on demonstrated reasoning in an authentic evaluation and decision-making scenario. To gain a more comprehensive understanding of teachers' digital competence, future research could incorporate classroom observations to investigate actual performance and technology integration during instructional practice. Such studies would provide valuable insights into how digital competencies are enacted in real-time teaching contexts.

## Data Availability

The raw data supporting the conclusions of this article will be made available by the authors, without undue reservation.
