# Supplementary material for: The impact of teacher training on the evaluation and selection of STEM augmented reality applications and TPACK self-assessment
Source: Front Psychol. 2025 Dec 16;16:1657028. doi: 10.3389/fpsyg.2025.1657028 (PMC12750621; doi:10.3389/fpsyg.2025.1657028)
Supplement: Supplementary file 1 [file Supplementary_file_1.docx]

Supplementary Material

# Prompts for the evaluation of the augmented reality applications

It should be noted that the prompts were translated into English for the purposes of this article, whereas the study itself was conducted in German.

| Imagine you are preparing a teaching unit on electric circuits/blood groups and heart/spatial geometry, and you come across two augmented reality applications related to this topic. You are about to test and evaluate both applications. We are interested in your assessment based on your pedagogical content knowledge and your technological pedagogical knowledge. You will proceed in five steps: 1. Initial assessment of the applications 2. Selection of one application under ideal conditions (e.g., availability of tablets and accessories, appropriate class level, etc.)  3. Description of a teaching unit using the selected application under ideal conditions (e.g., availability of tablets and accessories, appropriate class level, etc.)  4. Selection of one application under real-world conditions (in your everyday teaching context) 5. Description of a teaching unit using the selected application under real-world conditions (in your everyday teaching context) There is no character limit in the response fields. |
| --- |
| Test the augmented reality application using the accompanying materials and make an initial assessment. Write in full sentences what you observe about the application based on your pedagogical content knowledge and your technological pedagogical knowledge. Pay particular attention to features of cognitive and motivational learning support. We are interested in all your observations, whether positive, negative, or neutral. Please provide reasons for your comments where appropriate. |
| If you had to choose one of the two augmented reality applications under ideal conditions (e.g., availability of tablets and accessories, appropriate class level, etc.), which one would you select? Please explain your decision in full sentences. |
| Describe in full sentences and as much detail as possible how you would structure a lesson using the selected application under ideal conditions (e.g., availability of tablets and accessories, appropriate class level, etc.). |
| Consider which of the two augmented reality applications you would use under real-world conditions (in your everyday teaching context). Justify your choice in full sentences. |
| You would use application(s) X under real-world conditions (in your everyday teaching context). Describe in full sentences and as precisely as possible how your lesson planning under real-world conditions (in your everyday teaching context) differs from the lesson unit described above under ideal conditions. |

# Category system with content description and example

It should be noted that the category system was translated into English for the purposes of this article, whereas the study itself was conducted in German.

|  | Category | Content description | Example |
| --- | --- | --- | --- |
|  | 1. Contextual Knowledge (XK) | Knowledge about general framework conditions of teaching at the micro (learners), meso (school), and macro level (educational policy and society), which teachers cannot directly influence (e.g., availability of technical equipment, classroom space, or financial resources) | “Usability in school is a question, as no school where I have taught so far had tablets for the students (and budget problems do not give rise to any hope either) (…)” (315; pos. 17) |
|  | 1.1. Contextual Knowledge at the Micro Level | Knowledge about learners’ prerequisites, particularly regarding the use of technologies and materials | “Enough students have an Android smartphone so that they can work in groups of 2 to a maximum of 3 for sure.” (274; pos. 23) |
|  | 1.2. Contextual Knowledge at the Meso Level | Knowledge about school-specific framework conditions, especially regarding the availability of technical devices, materials, classroom space, budgets, and the school’s vision or strategy for using them | “It depends entirely on whether I have a device available on which I can download the app. We don't currently receive an Android device from the school.” (287; pos. 19) |
|  | 1.3. Contextual Knowledge at the Macro Level | Knowledge of the framework conditions shaped by educational policy and societal developments, particularly regarding the role that technologies currently play in curricula and policy agendas, as well as an understanding of broader megatrends such as mediatization and automation, which are driving significant transformations across all areas of society (e.g., evolving professional demands) | “The world is increasingly becoming digital (...).” (130; pos. 12) |
|  | 2. Technological Knowledge (TK) | Knowledge of various relevant digital media and technologies (e.g., notebooks or tablets). This includes both application knowledge (knowing how to operate devices) and conceptual background knowledge (understanding how digital technologies function, e.g., the structure of a computer) | “Easy to use, mistakes are virtually impossible as the app shows you step by step what to do. If there are any questions, there is a question button that provides additional information.” (162, pos. 15) |
|  | 2.1. Application Knowledge | Knowledge about operating technical devices such as notebooks or tablets, including installing programs, using and creating content (e.g., videos, websites), and solving technical problems | “The app can analyze any QR codes very quickly and it works very well to recognize the circuit.” (123; pos. 12) |
|  | 2.2. Conceptual Background Knowledge | Understanding how digital technologies work, such as the structure of a computer, different data structures, or the possibilities and limitations of algorithmic data processing. | “(AR, not to be confused with VR…)” (408; pos. 14) |
|  | 3. Technological Content Knowledge (TCK) | Knowledge of how technologies are used within the background disciplines of a school subject and how they influence or transform the discipline (e.g., imaging techniques in medicine). This also includes understanding the possibilities and limitations of specific technologies (such as the application) in relation to the subject content | “There are only two sockets and one double socket for the whole setup. So, you can't measure the voltage and current at several points without having to reinstall sockets and, if necessary, rescan the circuit.” (280, pos. 17) |
|  | 3.1. Knowledge about Technologies in Background Disciplines | Understanding how technologies are employed in the background disciplines of the school subject for generating, representing, using, and communicating knowledge, and how these technologies reshape the discipline (e.g., imaging techniques in medicine) | “The use of modern technology is practiced in a physical context; this connects to the real world, since certain professions also use tablets and similar devices to measure electrical circuits.” (147; pos. 15) |
|  | 3.2. Knowledge about the Possibilities of Technologies in Relation to Subject Knowledge | Knowledge about the potential and constraints of technologies (here specifically the application) as they relate to the subject content | “The paper circuit, on the other hand, makes it possible to try out different switch configurations in a time-saving way, which is a good way of demonstrating an alternating circuit, for example. The measuring device can be used to check the voltage at various points (yes/no).” (418; pos. 19) |
|  | 4. Technological Pedagogical Knowledge (TPK) | Knowledge about the use of media in teaching and media psychology (in the sense of "learning with media") as well as media education (in the sense of "learning about media") | "All the technical gimmicks distract from the actual learning content and quickly overwhelm students, as they cannot concentrate on the learning object but must deal with technical challenges." (183; pos. 11) |
|  | 4.1. Knowledge about the Use of Media in Teaching and Media Psychology | Knowledge about the use, design, and impact of digital media in teaching and learning contexts ("learning with media"). | "The app is easy to use and clearly designed, making it ideal for students and classroom use." (127; pos. 12) |
|  | 4.2. Knowledge about Media Education | Knowledge about the design possibilities of media (texts, images, etc.), media influences (e.g., on value orientations), and the conditions of media production and distribution (e.g., legal and economic aspects) ("learning about media") | "Promotion of digital work through the app." (160; pos. 15) |
|  | 5. Pedagogical Knowledge (PK) | Knowledge of general, subject-independent principles of teaching and classroom organization | "I would design one sequence for individual work and a second for group work so that the students can exchange information and share their experiences." (13; pos. 30) |
|  | 5.1. Knowledge about Learning Processes | Knowledge about how students learn, understanding cognitive, motivational, and emotional processes involved in learning | "Curiosity arises about what will happen when the power supply is turned on. This helps learners focus on the learning content = the electric circuit. Curiosity creates joy in further exploration." (418; pos. 12) |
|  | 5.2. Knowledge about Learner Diversity | Knowledge about the diverse individual differences in students’ abilities, backgrounds, and learning styles | "With larger circuits, especially those with 'gaps', assembling them neatly can be tricky; for students with fine motor difficulties, smaller ones might be more suitable." (315; pos. 16) |
|  | 5.3. Knowledge of Teaching-Learning Methods and Concepts | Knowledge of different pedagogical approaches, techniques, and methods for effective teaching and learning | "You can make decisions on your own and work at your own pace. You decide yourself whether and which cards to exchange." (154; pos. 28) |
|  | 5.4. Knowledge about Teaching and Learning Objectives | Knowledge of learning goals, including taxonomies and categories of learning objectives | "This would also show the teacher how well the students have understood the topic, because it corresponds to a relatively high taxonomic level according to Bloom." (122; pos. 20) |
|  | 5.5. Knowledge of Managing Learning Groups | Knowledge of organizing and guiding learning groups to create an effective learning environment | "As a lot of students’ responsibility is expected, the teacher should give clear instructions especially at the beginning." (130; pos. 13) |
|  | 5.6. Knowledge about Communication and Interaction with Learners | Knowledge of effective communication and supportive interaction to foster a positive learning environment | "I take on a coaching role, give feedback, and ensure good collaboration within the groups." (self-created example) |
|  | 5.7. Knowledge about Designing Learning Environments | Knowledge about creating physical and virtual learning spaces and designing materials to support the learning process and encourage learner engagement | "Using the app is easier or harder depending on the space available. If there's little room, it's difficult to rotate the figure since that’s not so easy to do with the app alone. But if the teacher ensures there is enough space, the additional booklets can be easily incorporated." (179; pos. 13) |
|  | 5.8. Knowledge about Individual and Learning Process Diagnostics | Knowledge of analyzing individual learning processes, including performance, motivation, or emotion assessments | "Students write a conclusion – good – neutral – negative – each group uploads their document to OneNote." (408; pos. 14) |
|  | 6. Content Knowledge (CK) | Knowledge about the subject matter itself (concepts, theories, etc.) | "Only technical current direction is shown. In reality, it should be the other way around." (317; pos. 14) |
|  | 6.1. Facts: Verbalizable Knowledge | Verbalizable and domain-relevant knowledge (i.e., “knowing that...”) | "The measuring device for voltage (V) uses only the term ‘electricity’, which is a rather unclear term" (137; pos. 17) |
|  | 6.2. Skills: Implicit Action Knowledge | Implicit, i.e. non-verbalizable action knowledge, from basic behaviors (pronunciation) to complex routines and action patterns (in the sense of "knowing how...") | "The measuring device can be connected 'wrongly' and then shows negative readings." (165; pos. 15) |
|  | 6.3. Concepts: Connected Conceptual Knowledge | Highly interconnected conceptual knowledge, which may be both verbalized and implicit (i.e., “knowing why...”) | "Current direction and electron flow are not distinguished. The pipe-and-water representation might give the impression that the water corresponds to the electrons (due to the flowing dots)." (182; pos. 14) |
|  | 7. Pedagogical Content Knowledge (PCK) | Knowledge about teaching and learning in the specific subject (conveying content knowledge, creating learning opportunities for skill development) | "Students already need some prior knowledge to measure voltage and current. → They need to know where (and why) to place the voltmeter and ammeter in the circuit for it to work." (280; pos. 16) |
|  | 7.1. Knowledge about the Potential of Tasks | Knowledge about cognitive demands, implicit prior knowledge requirements, didactic sequencing, long-term curriculum planning, and a task’s potential contribution to successful students’ knowledge construction | "It explained and illustrated the basic principles of electric circuits using familiar mechanisms in a very clear way. This helps in understanding what resistance does or what it means when there are branches, etc." (154; pos. 32) |
|  | *7.1.1. Requirement Level* | Knowledge about the requirement level of the task in relation to target grade levels or individual students (e.g., for differentiation), and alignment with textbooks or curriculum | "The app 'Insight Heart' is very complex and challenging to operate, presents content without a clear task, and doesn’t separately depict the core processes emphasized in lower secondary education. Thus, the information offered goes beyond regular instruction." (287; pos. 16) |
|  | *7.1.2. Required Prior Knowledge* | Knowledge about the subject-related prior knowledge students need for using the application and ideas for building that knowledge beforehand | "Before working with this app, I would ensure that the class has a grasp of the basics (concepts like resistance or Ohm, and the symbols used in a circuit – e.g., for a light source)." (162; pos. 20) |
|  | *7.1.3. Learning Content* | Knowledge about what students can (or cannot) learn using the app and the range of topics/activities it is suited for | "I think AR circuit constructor is great for exploring the basics of resistors. However, I see that AR paper circuits, when used properly, can cover the whole topic of circuits (resistors, switches, loops, amperes, volts, etc.) since the components can also include, for example, a voltmeter." (202; pos. 32) |
|  | 7.2. Knowledge about Student Cognitions and Diagnosing Student Knowledge | Knowledge of subject-related students’ cognitions (misconceptions, typical errors, difficulties, strategies, ideas and beliefs) as well as the diagnosis of students’ knowledge (e.g. including prior knowledge) and comprehension processes and appropriate responses to students’ answers and errors | "This kit is very similar to possibly outdated kits already in use, which leads to students experimenting very cautiously because ‘you can easily break things with electricity.’" (318; pos. 14) |
|  | *7.2.1. Interest* | Knowledge about students’ interests and motivation related to the subject | "Some students would probably prefer to experiment with 'real' circuit boxes because they may already be familiar with them (from home, their father’s job, etc.) or like to try things delicately." (162; pos. 17) |
|  | *7.2.2. Typical Difficulties and Errors* | Knowledge about common errors and difficulties students have with this topic and how to respond appropriately | "Without an introduction, students might set the voltage too high and burn out the bulbs." (317; pos. 13) |
|  | *7.2.3. Student Conceptions* | Knowledge about students’ pre-, during-, or post-instruction conceptions and how to appropriately address them | "I also really like that it becomes visible (even tangible) that the donation concerns red blood cells (and not antibodies). This is often a misconception among students, which the app’s representation can correct." (287; pos. 9) |
|  | *7.2.4. Diagnosing Student Knowledge* | Knowledge about diagnosing students subject-related prior knowledge and understanding processes | "Differences only become apparent when students are confident enough in their knowledge to want to build larger or more complex circuits and then experiment with current and other metrics or measuring devices." (318; pos. 23) |
|  | 7.3. Knowledge about Making Content Understandable | Knowledge of subject-specific representational activities and teacher interventions to support students’ active knowledge construction | “As with any model, you have to address the fact that this is a mental model and does not represent reality (electricity does not fall from a higher point to a lower point – waterfall model).” (122; pos. 13) |
|  | *7.3.1. Model Competence* | The ability to use models to gain purpose-related knowledge and to make judgments about models with reference to their purpose, the ability to reflect on the process of gaining knowledge through models and modeling, and the willingness to apply these skills in problematic situations | "I personally find the open water circuit most suitable to argue voltage difference using height difference – this is very well illustrated with the app." (78; pos. 25) |
|  | *7.3.1.1. Model Knowledge and Use* | - Model knowledge: Knowledge of basic models that provide learners with access to the corresponding model-based concepts of the subject content in the sense of sound specialist knowledge  - Model Use: Using models in knowledge-building processes and the ability to develop models independently | "I’m personally a big fan of the bicycle chain model, but it reaches its limits in concepts like parallel circuits – which needs to be communicated to students." (260; pos. 26) |
|  | *7.3.1.2. Model Understanding* | Knowledge about the importance of models and their use (superordinate dimension), part of an adequate Nature of Science concept (role and nature of models) | "None of the models (models = simplified versions of reality) can correctly represent all properties of an electric circuit. Therefore, it is enriching to use and compare different models." (344; pos. 12) |
|  | *7.3.2. Visualization* | Knowledge about visualizing subject content | "There is a direct link between electric circuits and their symbols (how they should be drawn) and the actual circuit with the bulb and so on." (122; pos. 15) |
|  | *7.3.2.1. Representation Forms* | Knowledge about forms of representation, i.e. the representation of a learning object in three different ways (enactive, iconic, symbolic) | "The representation on the tiles is the same as in a plan. A connection can be made between the plan and reality. Conversely, a real-life representation can lead to the plan – from enactive to iconic." (132; pos. 15) |
|  | *7.3.2.2. Multiple Representations* | Knowledge of the importance of multiple representations (different forms of representation) to effectively convey complex concepts | "There are several types of visualizations (bicycle chain, water circuit with turbines, or waterfall model)." (122; pos. 12) |
|  | *7.3.2.3. General Visualization Cues* | Knowledge of how to visually represent subject matter effectively | "I see a brown vein as the site of dead myocardium during a heart attack. This can lead to misconceptions." (243; pos. 14) |
|  | *7.3.3. Explanations* | Knowledge of quality aspects of explanations in subject content | "A brief explanation of what exactly happens during blood donation would have been useful for further understanding. You can see different results for compatibility and incompatibility, but it’s not explained why. You can only learn which blood groups are compatible, but not why or what exactly happens." (19; pos. 24) |
|  | *7.3.4. Real-Life Connection* | Knowledge about the relevance of real-world contexts for representations, tools, and instruction | "Still, the 'real' handling of electricity is missing, which would allow safety aspects to be directly addressed." (365; pos. 15) |
|  | *7.3.5. Lesson Planning and Guidance* | Knowledge about planning and accompanying lessons in subject teaching | "In the topic of the human body in grade 7, I would first cover blood in general – the circulatory system, blood composition, etc. Then, I’d plan a sequence where students already know about blood donation and that not everyone can donate to just anyone due to different blood groups." (237; Pos. 17) |
|  | 8. Technological Pedagogical Content Knowledge (TPACK) | Knowledge about how specific technologies can be meaningfully used in subject teaching | "The cognitive activation, especially when changing perspective and the spatial presentation of the cracks, add clear value to the lesson, especially in spatial geometry. The pacing would be different and the playful and motivating aspect of the other 'dry exercises' would definitely persuade me to use the first app. Previous teaching units were based on practical exercises with a number cube or soma parts and LEGO bricks, but I would still keep them." (406; pos. 17) |
|  | 8.1. Knowledge about the Potential of Tasks Using the Medium | Knowledge about how tasks using the medium can contribute to successful students’ knowledge construction | "AR circuit constructor provides greater added value for me. While AR paper circuits simulate electrical components very well, I can easily replicate their functions physically. On the other hand, it’s much harder to illustrate a water circuit model like a circuit diagram in a way that students benefit visually. Just for explaining the concept of a circuit, AR circuit constructor is very illustrative, although pitfalls must be addressed." (260; pos. 32) |
|  | 8.2. Knowledge about Student Cognitions and Diagnosing Student Knowledge Using the Medium | Knowledge of subject-related student cognitions (misconceptions, typical errors, difficulties, strategies, ideas and beliefs) as well as the diagnosis of student knowledge (including prior knowledge) and comprehension processes and appropriate responses to student responses and errors in connection with the medium | "Some students might struggle to transfer the digital circuit to a real-world representation of a complex circuit. That’s why I wouldn’t rely solely on this app but use it for visualization and practice." (162; pos. 17) |
|  | 8.3. Knowledge about Making Content Understandable Using the Medium | Knowledge of specific subject-related presentation activities and intervention possibilities of the teacher with the medium, which serve to support the active knowledge construction processes of the students | "I would start with current and series circuits using the bicycle model. Students observe what happens when a single bulb is added and then a second in series. They first try to explain this using the bicycle model (in the app), and then we discuss it together. Then I would explore voltage using the open water circuit model. Again, we’d look at a series connection with lights (without the app), then with the app. The explanation follows. The teacher asks what happens in a parallel connection; students observe the bulbs and try to explain it with the water circuit model." (78; pos. 33) |
